# Editorial: The Impact of AI-Enabled Technologies in E-commerce and Omnichannel Retailing

**DOI:** 10.3389/fpsyg.2021.718885

**Published:** 2021-07-08

**Authors:** Monica Cortinas, Carmen Berne, Raquel Chocarro, Frode Nilssen, Natalia Rubio

**Affiliations:** ^1^Institute for Advanced Research in Business and Economics, Public University of Navarre, Pamplona, Spain; ^2^Department of Marketing Management and Market Research, University of Zaragoza, Zaragoza, Spain; ^3^Nord University Business School, Bodø, Norway; ^4^Department of Finance and Marketing Research, Business Studies Faculty, Autonomous University of Madrid, Madrid, Spain

**Keywords:** information and communication technologies, artificial intelligence, consumer's behavior, retailing, adoption of technologies

This Research Topic aims to understand the impact AI technologies are having on the online retail market. The topic includes four papers, two of them focusing on the process of adoption of technologies by firms and another two from the consumers' point of view, therefore providing an excellent general perspective of the phenomenon.

Organization's adoption of technology has been an important topic over decades. There is also little doubt that technology influence and has an impact on how organization work and to some extent also on how they are organized. Various disciplines have addressed this issue and many perspectives have been applied over time. The rapidly growing interest for studying technology adoption strategies and outcomes of the implementation of these strategies have led to a wide range of contributions across disciplines. The richness of contributions illustrates the need for knowledge development in this field. At the other side, there is also time and need for some consolidation and review for future directions of the research effort. Saghafian et al. of the article “Stagewise Overview of Issues Influencing Organizational Technology Adoption and Use” provide a thematic map of the existing literature consisting of different theoretical frameworks, findings, and suggested interventions in one stagewise overview. The paper provides both a systematic comparison and presentation of various perspectives, as well as inspiration to future research in the field.

The second paper, by Villarejo-Ramos et al., presents a pioneering study that identifies determinants (drivers and barriers to use) of companies' adoption of Big Data Applications. The authors extend the UTAUT model, including the influence of the resistance to use and the perceived risk. Further, they perform a neural network prediction. Behavior, socioeconomic factors, and user experience represent the biggest impact on the intention to use the Big Data system. From 199 observations of Spanish companies (46.23% used Big Data for commercial purposes), the results show that the acceptance and use of big data will be enhanced if they believe that the tool improves their performance or if they see that other companies in their environment use it. In contrast, the use of Big Data can be held back by cultural factors and those related to skills within the organization and the perceived risks related to their use.

The following two papers focus on the adoption of these technologies by consumers. In the third paper, Romero et al. contribute in two ways to the understanding of effects of AI-enabled technologies, particularly Voice-Activated Personal Assistant (VAPA) incorporated in smart speakers, in the intention to visit an establishment. On the one hand, the paper advances in the analysis about how the engagement process evolves between humans and smart speakers, particularly how such engagement influences the customer attitude and intention toward the recommendations of a store, brand, or firm. On the other hand, it analyzes how two features of the VAPAs' recommendations (namely gender congruence between the customer and the voice in the device and the amount of information in the message) influence the effectiveness of such recommendations. Their results show that that gender congruence generates greater user engagement with the smart speaker and the message length is positively related to attitudes toward the establishment.

Finally, the fourth article by Marín-García et al. analyzes the effect of Information and Communication Technologies (ICT) on the customers' perceptions of innovation and sustainability practices adopted by the retailers and the moderating role of store formats. ICT are revolutionizing the relationship between firms and customers with the growing implementation of AI technologies in customer services. Retailers are concerned with incorporating these innovative tools in their daily activities to improve their image, better satisfy their customers, retain them and consequently be more competitive. The authors show that the degree of technological advancement of retailers positively affect their innovative and sustainable actions for the three store formats examined (hypermarkets, supermarkets, and discounts) and that the customer association for the use of ICT and innovative practices are especially intense in hypermarkets and supermarkets compared to discounts stores. The research concludes that ICT, innovation, and sustainability are strongly related, being ICT the tools that make innovation and sustainable tangibles for the customers' eyes.

The Research Topic provides a general overview of the critical changes that AI and ICT continue to provide for firms and consumers. As Brynjolfsson and Mcafee (2017) note, AI is probably the most important general-purpose technology in our era. As such, it can affect the entire economy through waves of complementary innovations and opportunities. However, research on the consequences of this technology on business activities, and its interaction with consumers is still scarce and only partially addressed. This topic contributes to the understanding of these consequences from the dual point of view of firms and consumers, showing the different potential ways by which AI technologies, ICT and innovation add value to customers and firms.

## Author Contributions

CB, RC, FN, and NR have summarized the contribution of one paper. MC has reviewed those summaries and compiled the final note. All authors form part of the editorial team.

## Conflict of Interest

The authors declare that the research was conducted in the absence of any commercial or financial relationships that could be construed as a potential conflict of interest.
